# X-ray techniques for innovation in industry

**DOI:** 10.1107/S2052252514021368

**Published:** 2014-10-31

**Authors:** Krystyna Lawniczak-Jablonska, Jeffrey Cutler

**Affiliations:** a Institute of Physics, Polish Academy of Sciences, Warsaw, 02-668, Poland; b Canadian Light Source, 44 Innovation Blvd, Saskatoon, Saskatchewan S7N 2V3, Canada

**Keywords:** X-ray techniques, industry, innovation

## Abstract

The smart specialization declared in the European program Horizon 2020, and the increasing cooperation between research and development found in companies and researchers at universities and research institutions have created a new paradigm where many calls for proposals require participation and funding from public and private entities. This has created a unique opportunity for large-scale facilities, such as synchrotron research laboratories, to participate in and support applied research programs. Scientific staff at synchrotron facilities have developed many advanced tools that make optimal use of the characteristics of the light generated by the storage ring. These tools have been exceptionally valuable for materials characterization including X-ray absorption spectroscopy, diffraction, tomography and scattering, and have been key in solving many research and development issues. Progress in optics and detectors, as well as a large effort put into the improvement of data analysis codes, have resulted in the development of reliable and reproducible procedures for materials characterization. Research with photons has contributed to the development of a wide variety of products such as plastics, cosmetics, chemicals, building materials, packaging materials and pharma. In this review, a few examples are highlighted of successful cooperation leading to solutions of a variety of industrial technological problems which have been exploited by industry including lessons learned from the Science Link project, supported by the European Commission, as a new approach to increase the number of commercial users at large-scale research infrastructures.

## Abstract 2

Are synchrotrons needed for innovation in industry? What can scientists at large-scale facilities offer for R&D in industry? Is the comfort of life profiting from research?

## 1. Introduction   

Since the early development of dedicated synchrotron research facilities in the 1960s, industrial research has found a need for the unique tools available and some of the early technology adopters were from the private sector including Farrell Lytle from the Boeing Corporation, one of the godfathers of extended X-ray absorption fine-structure (EXAFS) spectroscopy, who in 1971 published a paper with Ed Stern and Dale Sayers on EXAFS spectroscopy (Sayers *et al.*, 1971 ▶). Three years later, in 1974, Lytle and Sayers published work on the characterization of heterogeneous catalysts which opened the door to industrial utilization of various X-ray absorption techniques (Lytle *et al.*, 1974 ▶). Since 1974, X-ray absorption spectroscopy (XAS) applications have grown and touched on a broad spectrum of industry sectors, which is reflected in the open literature and patents. Patents were found to cover a broad spectrum of industry sectors including catalysts, engineering, energy and many consumer products.

Globally, research and innovation pre-eminence are seen as a competitive advantage for industries including aerospace, automotive, manufacturing, agriculture, mining, health, nanotechnology and green energy. This is reflected in the gross domestic expenditures on research and development (GERD) as reported by the Organization for Economic Co-operation and Development (OECD). In 2012, OECD members reported that GERD was slightly over 1 trillion euro [on average, 2.4% of gross domestic product (GDP)], with about 60% conducted by the private sector; in the European Union-15, over 300 billion euro (2.2% of GDP) was spent on R&D, with ∼40–65% of that research conducted by industry (OECD, 2014 ▶).

With the large investment made by the private sector in innovation, industry appreciates that leading-edge research and development is crucial to future prosperity and wealth creation and can only be accomplished through collaboration and partnerships. Critical to such a culture of innovation are the small- and medium-sized enterprises (SMEs) which are often seen as the drivers of economic prosperity and growth. Scientists understand that in many calls for scientific projects cooperation with industry is a requirement and they are ready to participate in such projects by offering their expertise and knowledge in areas such as fabrication and characterization of a novel material. The question arises: is industry ready to take advantage of these opportunities? The large multinational companies which have their own in-house R&D expertise have historically collaborated with scientists outside their organizations, particularly at large scale facilities (LSFs) such as synchrotron laboratories which bring new tools to industrial dilemmas. By developing an understanding of individual industry sectors, the scientists at LSFs and other academia and research institutions are able to collaborate with key partners, as well as develop scientific expertise in several industry-relevant areas. This allows the scientist to work directly with the client to identify questions and solutions within each sector, and to determine the best techniques to apply to the problem.

### 1.1. Industrial needs and synchrotron solutions   

Synchrotrons are used by industry for a number of reasons. For many industrial problems, the unique properties of synchrotron light allow research to be conducted that is impossible by other analytical means. Synchrotron science is an economical research tool because of the clarity of the research results and the speed with which the sample analysis can take place. Synchrotrons and other large-scale infrastructure are multidimensional research centres with research infrastructure and scientific staff that can support a number of different industrial sectors such as: soil and environmental science, atmospheric chemistry, catalysis, tribology, battery materials, nanomaterials, macromolecular crystallography and biomaterials.

This combination of resources and scientific expertise gives these facilities the opportunity to be a solution and service provider in the analytical services sector.

In order to support the various needs of industrial clientele, scientists will often work with industry staff to provide a range of services, including:

*Analytical services* – these are quick turn-around projects with typical timelines of six months or less and very narrow scope. The projects that would tend to be identified as analytical services include the purchase of beamtime for pharma and automotive or aerospace system support, or using X-ray absorption spectroscopy, X-ray diffraction and scattering, and X-ray-computed microtomography for the chemical speciation of metals in mining or sampling of new materials.

*Collaborative research* – these are typically longer term projects which may bring one or two partners together for a specific study in order to realise a shared goal. These projects will tend to have timelines of two to three years and include projects with industry and academic partners.

*Strategic research* – these are longer term projects which will bring multiple partners together from academia, government and industry to work on a common activity and are often larger-scale research programs which pool resources in order to achieve a common goal. These programs tend to have longer time lines, of the order of three to five years.

Collaborations and strategic research programs have often led to significant technology development that has been exploited by industry partners.

With a significant amount of innovation coming from SMEs, many industry project ideas are raised by these small- and medium-sized enterprises which are either not aware of the capabilities of synchrotron facilities and how they can help, do not have enough cash flow to conduct research projects, or do not have the expertise that will allow them to take advantage of these research opportunities. With SMEs being such an important economic driver, it is important for governments to answer the following question: What can be done to change this situation and enable SMEs to participate in technology development?

**Figure 1:**
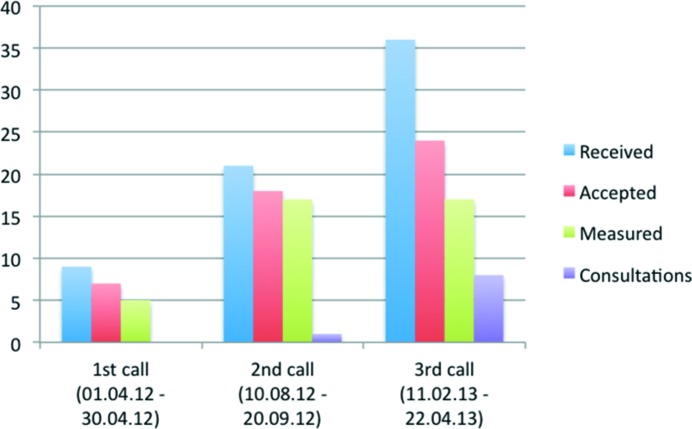
Number of industry proposals received in Science Link from the three proposal calls (blue), accepted proposal (red), performed (green) and consulted for laboratory experiments (violet).

One such answer to the question is the Science Link project (Photons and Neutrons for Innovation, Economic Growth and Entrepreneurship in the Baltic Sea Region), a flagship project of the European Union Baltic Sea Region program, whose main goal is to support and encourage innovation and entrepreneurship in the Baltic Sea Region (BSR) by offering industrial customers free access to techniques available at BSR large-scale infrastructures such as the MAX IV (Lund), HZB (Berlin) and DESY (Hamburg) synchrotrons. To achieve this mandate, project Science Link covers the research costs of SMEs and offers them free access to LSFs; moreover it provides LSFs with personnel to take care of industrial clients. In order to build awareness of the program and to help SMEs to make optimal use of the facilities, it was necessary to establish local contact points to closely collaborate with industry and academic partners (universities and R&D institutes). Nine regional agencies have the mandate of working with SMEs to explain the Science Link program and how it will help them to be more innovative, and to encourage SMEs to apply for a research program. As a result of the first three calls for industry proposals since 2012, 66 industrial projects have been submitted covering many disciplines (Figs. 1 ▶ and 2 ▶). Of note, most of the project proposals specified a technological problem and have been seeking a solution. In 16 of the proposals, it was sufficient to apply conventional laboratory techniques to solve the problem. Out of the original 66 projects, 48 projects were awarded experimental time at LSFs. A single experiment may not solve the technological problem proposed by the SME but indicate a possible solution; the expectation being that the company will pay for the next service. Indeed, in a few cases, the projects have continued on a commercial basis. After three rounds of proposals, the main lesson learned from the Science Link project is the need for direct contacts with the SMEs and easy access to an explanation of the problems which can be solved by a given technique by:

(*a*) identifying specific companies with potential synchrotron usage needs,

(*b*) contacting and establishing relationships with research staff and managers who can facilitate projects,

(*c*) identifying specific projects where synchrotron analysis (or other LSFs) can be valuable to the company,

(*d*) conducting demonstration projects at an LSF,

(*e*) completing data analysis, interpretation and reporting to the company,

(*f*) conducting projects within a business environment of confidentiality agreements and contracts.

From the sophisticated description of the techniques at LSFs, it is often difficult for managers working in the private sector to see the connection between the tools found at LSFs and the solutions they are trying to find to their technological problems. In particular, SMEs are looking for scientific methods to solve their problems in a timely fashion and at a low cost. Therefore, more work needs to be done at scientific institutions to develop the means to help industry, in particular SMEs, to understand the capabilities of LSFs through specialized training, demonstration projects, and better communication and marketing tools. Awareness and capacity building will be crucial to the future utility of LSFs by SMEs. In this paper, we highlight several examples of scientific activity conducted at various synchrotron light sources in partnership with industrial partners.

## 2. Examples of industrial projects studied with synchrotron techniques   

Specific examples of industry-sector-focused projects are described below.

### 2.1. Structural and chemical properties of double metal cyanide catalysts   

Double metal cyanide (DMC) catalysts are commonly used for industrial ring-opening polymerization of the epoxides, which is an initial stage in the manufacture of polyurethanes (Chruściel *et al.*, 2014 ▶). This group of catalysts has been successfully used and progressively developed for decades, but knowledge on the molecular nature of their particularly high activity and selectivity is limited to some phenomenological hypotheses based on overall chemical premises. To shine some light on the relationship between structural and chemical properties of DMC catalysts and their activity, XAS studies were performed in cooperation with the MEXEO Kędzierzyn-Koźle Company. The catalyst (which has the trade name DMC–MEO) and the reference material were synthesized by MEXEO. The reference material was a hydrated trizinc bis[hexacyanocobaltate(III)] compound (Zn_3_[Co(CN)_6_]_2_·*n*H_2_O) of very low catalytic activity without practical application. Only after introduction to its structure of an appropriate organic ligand does the catalytic activity increase. In the investigated sample, the *tert*-butanol (*^t^*BuOH) ligand was introduced in a technological process. This ligand is frequently used in the commercial application of the DMC family of catalysts. The commercial DMC catalyst was used as the comparative DMC catalyst.

**Figure 3:**
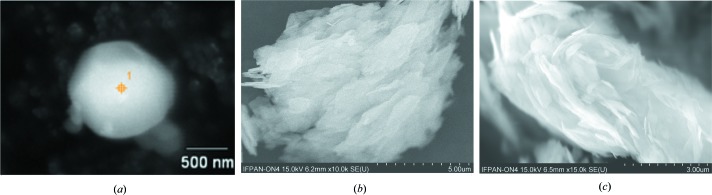
SEM images of (*a*) reference material, (*b*) DMC–MEO catalyst, (*c*) reference commercial DMC catalyst. The introduction of a ligand changes the morphology of the powder from cubic, observed in reference material (*a*), to irregular sheets with a very extended surface (*b* and *c*).

SEM images showing the morphology of the powder materials are presented in Fig. 3 ▶. The introduction of the ligand changes the morphology of the powder from the cubic structure observed in the reference material to irregular sheets with a very extended surface. The size of grains in the analyzed powders varied from 0.5 to 10 µm.

EXAFS analysis of the Zn and Co *K*-edges was performed to examine the local atomic order around the Zn and Co atoms in the reference material, the DMC–MEO catalyst and the commercial catalyst. The XAS measurements were performed at SOLEIL, France (SAMBA station).

**Figure 4:**
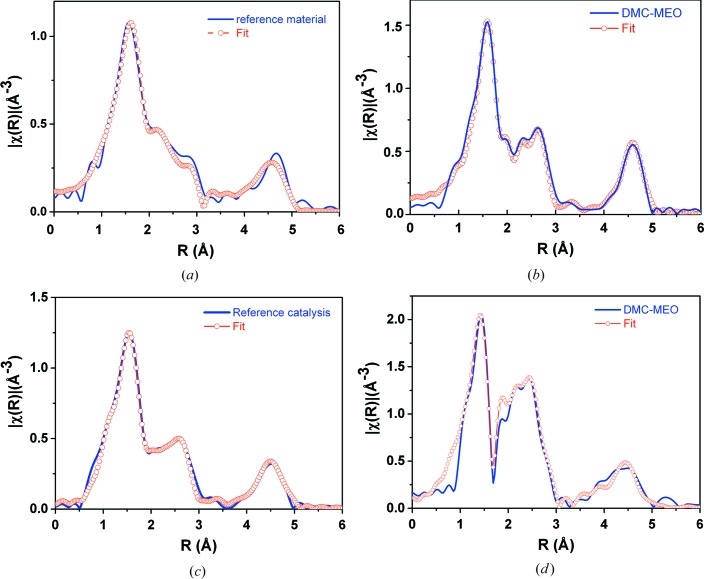
Fits to the Fourier transform EXAFS data of the model of the local atomic structure around Zn atoms at the Zn *K*-edge (*a*) reference material, (*b*) DMC–MEO catalyst and (*c*) DMC reference; and (*d*) around the Co atom–Co *K*-edge DMC–MEO catalyst.

In agreement with XRD results, the model for the reference material was assumed to be a cubic structure (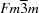) with water and a lattice constant of 1.0249 nm. This model suggested, for the EXAFS analysis, the number, type and distance of atoms in the subsequent coordination shells in the ideal (Zn_3_[Co(CN)_6_]_2_·12H_2_O) reference material. The EXAFS analysis (Fig. 4 ▶ *a*) indicated that in the considered reference material instead of 24 only 3 O atoms were detected around the Zn atom. In the other coordination spheres, the number of atoms was in the agreement with the model. For both catalysts, the number of atoms in the subsequent coordination spheres was smaller than in the model. These results suggested that the stoichiometry and the catalyst structure change in both catalysts. Several models of atomic order around Zn were considered for both catalysts. Finally, it was shown that the atomic order in these materials resembles a rhombohedral structure with four N atoms around Zn instead of six as in a cubic structure.

The best fit to the EXAFS data was for the model assuming that about 84% of Zn atoms in the DMC–MEO catalyst still have local atomic order as should be the case in the reference material with a rhombohedral structure but 16% have two Cl atoms in the first coordination sphere (Fig. 4 ▶ *b*). In the case of the commercial reference catalyst, 90% of Zn still has coordination as in the reference rhombohedral material and 10% of the Zn was bonded to Cl (Fig. 4 ▶ *c*).

The local atomic structure around the Co atom did not change in practice in all of the investigated materials (Fig. 4 ▶ *d*). This confirmed that the Co metallic centre is not active during the preparation of the catalyst.

The results of the XAS analysis support a model similar to that proposed by Kim *et al.* (2003 ▶), Zhang *et al.* (2007 ▶) and Wojdeł *et al.* (2007 ▶). In this model, the catalyst forms cluster-like complexes with the Co atomic structure not affected as compared with the reference rhombohedral material. The Zn atoms inside the clusters have also not changed atomic order. This explains that some of the Zn atoms in EXAFS analysis have an atomic order similar to that in the reference material. At the surface of the clusters, Zn atoms are bound partially with cyanide groups and Cl atoms. The amount of chlorine detected using EXAFS in all the investigated samples was in agreement with that estimated using energy-dispersive X-ray spectroscopy (EDS) and X-ray photoelectron spectroscopy (XPS) measurements. The results of the EXAFS analysis have been submitted for publication (Lawniczak-Jablonska *et al.*, 2014 ▶).

### 2.2. Comprehensive characterization of mineral ilmenite used in pigments production   

Ilmenites are valuable natural sources of titanium and titanium compounds, widely used in industry for the production of white pigments. The quantitative analysis of the phase content in the raw materials (TiO_2_) used for white pigment production is very important for the proper adjustment of chemical reaction conditions (Jablonski *et al.*, 2012 ▶). The composition of minerals can vary depending on the location where they are collected. Industrially exploited ilmenite deposits are situated in countries such as Australia, China, India and Norway. Owing to the substantial differences in the climate (temperature and humidity) and geological conditions (the nature of source rock and age of deposits) in the places of origin, differences in the chemical composition can be expected. In the process of TiO_2_ production by the sulfate method, reaction of titanium raw material with sulfuric acid is the first step, which is highly exothermic and should be properly adjusted to avoid explosion. Knowledge of elemental, chemical and phase composition has a large influence on the efficiency, safety, kinetics of reaction and the quality of products. The materials are largely inhomogeneous, and conventional diffraction and X-ray fluorescence microanalysis provide very inconsistent results which in many cases do not match with each other. This is why the content of elements measured by conventional electron probe microanalysis (EPMA) is usually given as a content of the most popular oxides (*e.g.* TiO_2_, FeO, SiO_2_), which does not fit to the real content of oxygen in the material and contents of particular chemical compounds. Besides FeTiO_3_ (the chemical formula of ilmenite), the raw material contains many other compounds (Klepka *et al.*, 2005 ▶).

The main phases identified in ilmenites were ilmenite (FeTiO_3_), hematite (Fe_2_O_3_), geikielite (MgTiO_3_), pseudorutile (Fe_2_Ti_3_O_9_) and enstatit (MgSiO_3_), as well as small quantities of kleberite [FeTi_3_O_6_(OH)_3_], MnTiO_3_ and CaSiO_3_. A higher content of the pure ilmenite phase was found in Norwegian ilmenite and a lower content in Chinese and Indian ilmenites. The lowest content of the pure ilmenite phase was detected in Australian ilmenite. In contrast, in the Australian ilmenite, a higher content of the pseudorutile phase was observed. In Chinese and Norwegian ilmenites, the pseudorutile phase was not detected but a significant content of hematite was present. In the case of other ilmenites, a considerably smaller content of hematite was observed (Jablonski *et al.*, 2012 ▶).

The minerals are highly heterogeneous; there are some grains that contain more than 70% of one detected phase. For detailed study of heterogeneous materials, a single particle approach in EPMA (Spolnik *et al.*, 2005 ▶) was found very reliable and useful (Klepka *et al.*, 2005 ▶).

**Figure 5:**
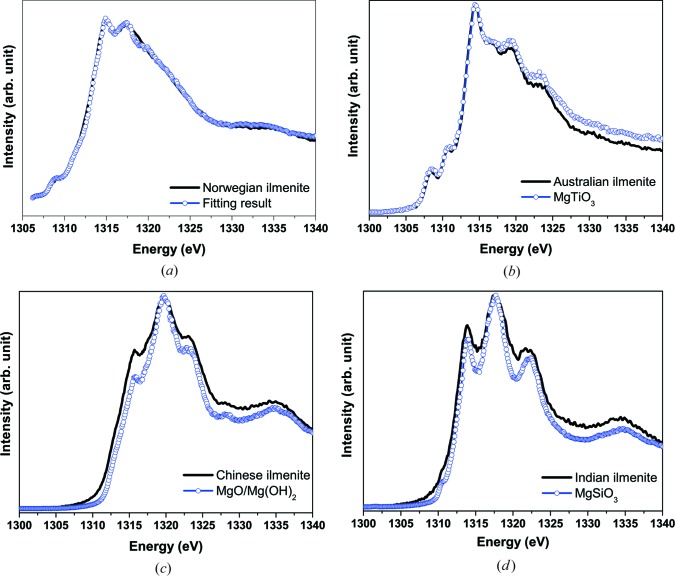
(*a*) Mg *K*-edge for Norwegian ilmenite (line) and result of fitting the linear combination of reference compounds MgTiO_3_ – 57.5%, MgSiO_3_ – 30%, MgO/Mg(OH)_2_ – 12%, (open circles); (*b*) Australian ilmenite and geikielite (MgTiO_3_); (*c*) Chinese ilmenite and a mixture of MgO/Mg(OH); (*d*) Indian ilmenite and enstatit (MgSiO_3_).

The chemical compounds based on the minority elements influence the quality of the final product and are difficult to identify. A knowledge of the chemical bonding of the minor elements enables ilmenite processors to properly adjust chemical reactions and to improve the quality of the final product. To identify the chemical compounds formed by Mg, Mn and Cr in natural ilmenites originating from different places, XAS was applied (Fig. 5 ▶) (Klepka *et al.*, 2010 ▶). The XAS measurements were performed at the BESSY II synchrotron, station UE52-PGM (*K*-edge of Mg) and DORIS, DESY Hamburg, station A1 (*K*-edge of Mn and Cr).

The most remarkable result was obtained for Norwegian ilmenite where Mg was found to exist in three chemical compounds [MgTiO_3_, MgSiO_3_ and probably a mixture of MgO/Mg(OH)]. Moreover, Mg forms different chemical compounds in each of the investigated ilmenites (Fig. 5 ▶). Therefore, it is clear that the chemical bonding of Mg depends on the climatic and geological conditions at the place of origin.

In the case of the other investigated elements (Mn and Cr), it turns out that the chemical compounds present are similar in all considered ilmenites, MnTiO_3_ for Mn and Cr_2_O_3_ for Cr. The only exception is the lack of Cr in Australian ilmenite. Therefore, the chemical bonding of these elements is driven rather by chemical affinity than climatic and geological conditions.

Thermokinetic investigations showed that the presence of an Fe_2_Ti_3_O_9_ phase has an influence on the reaction (Jablonski *et al.*, 2012 ▶). Significant differences were observed between curves of thermal power of Australian and Indian ilmenites where this phase is present and Norwegian and Chinese ilmenite where it is absent. The differences in thermokinetics curves between ilmenites with similar phase composition (Norwegian and Chinese) can be explained by differences in the content of individual phases and the presence of different phases of accompanying elements.

### 2.3. Activity of an antimalarial drug at the molecular level in solution studied using XAS   

**Figure 6:**
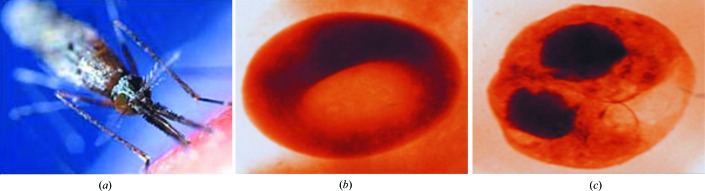
The formation of hemozoin at the molecular level is still not well understood. Images show (*a*) a mosquito (http://news.bbc.co.uk/2/hi/health/3690049.stm), (*b*) an uninfected human red blood cell and (*c*) a cell with malarial pigment (hemozoin) (courtesy of C. Magowan, W. Meyer-Ilse and J. Brown, LBNL, California, ALS, X-ray microscopy beamline).

Malaria remains the world’s most prevalent disease that causes severe health problems, particularly in African and Asiatic countries. Efforts to control malaria are fated unless a new approach can be found. The most recent reports from the World Health Organization point out that malaria killed more than 600000 people in 2012, with 90% of these deaths occurring in sub-Saharan Africa. An effective malaria vaccine does not exist at the moment and therapy is totally based on the use of preventative drugs. It is known that malaria parasites are susceptible to quinoline-containing and artemisinin drugs only at the time of hemoglobin degradation and production of malarial pigment. During its intraerythrocytic phase, the malarial parasite digests globin chains of the host cell’s hemoglobin. The remaining heme (ferriprotoporphyrin IX – FePPIX) causes lethal changes to membranes so its efficient disposal in the form of so-called malarial pigment or hemozoin crystalline material is of critical importance to the parasite (Fig. 6 ▶). The formation of hemozoin and the action of an antimalarial at the molecular level is still not well understood. The XAFS technique was applied to approach this problem. The results of these studies are presented in Walczak *et al.* (2005 ▶, 2010 ▶), Walczak, Lawniczak-Jablonska, Wolska, Sienkiewicz *et al.* (2011 ▶) and Walczak, Lawniczak-Jablonska, Wolska, Sikora *et al.* (2011 ▶).

The crystal structure of hemozoin has been solved using X-ray powder diffraction and its synthetic analogue, β-hematin, was synthesized by Bohle *et al.* (1997) ▶. The XAS method cannot be applied to infected blood samples treated with chloroquine (CQ) because of the significant amounts of iron appearing outside of the FePPIX–CQ complex, for example in the hemoglobin itself. Therefore, the basic studies are performed on model materials, which can be considered to be hemozoin synthetic substitutes. In the work by Walczak *et al.* (2005 ▶, 2010 ▶), it was shown that hematin anhydride (β-hematin) and mesohematin anhydride can be used as synthetic equivalents of hemozoin. Subsequently, the studies were concentrated on the possibility of the formation of a hemozoin-like FePPIX dimer–CQ complex. This kind of chemical association might be present in the digestive vacuole of a parasite. The ferriprotoporphyrin IX is believed to be a target for commonly used antimalarial drugs but their interactions are still not understood on a molecular level. The goal of the studies was to find the drug-induced perturbations of the structures of a soluble β-hematin-like compound [iron(III)-(*meso*-porphyrin-IX anhydride)] called mesohematin. The XAS measurements on a frozen sample of mesohematin in solution at the iron *K*-edge were performed at ESRF (station ID26). High-resolution XANES (X-ray absorption near-edge structure) and EXAFS spectra on the iron *K*-edge enabled us to reveal the differences in the local environment of Fe atoms inside the mesohematin structure in investigated solutions before and after the addition of the chloroquine drug. Special interest was drawn to the axial linkage between the central Fe atom of the FePPIX coordinated axially to the propionate group of the adjacent FePPIX. This kind of bonding is typical for hematin anhydride. Detailed analysis revealed differences in the oxygen coordination sphere (part of the dimer linkage bond) between synthetic equivalents of hemozoin in the powder state, and dissolved in acetic acid and water at different concentrations mimicking the physiological conditions of the parasite’s food vacuole. In the paper by Walczak, Lawniczak-Jablonska, Wolska, Sikora *et al.* (2011 ▶), it was shown that the molecular structure of the synthetic analogue of hemozoin is no longer dimer-like in acidic solution and in the presence of chloroquine. Such an effect was not observed in the solution with dimethyl sulfoxide (Walczak, Lawniczak-Jablonska, Wolska, Sienkiewicz *et al.*, 2011 ▶). The EXAFS analysis showed that the main effect of chloroquine action in the presence of water molecules is an increase in the amount of ferri-*meso*-porphyrin IX molecules with ferric iron and without the stable axial iron–ligand bonding. Therefore, the tested antimalarial drug, chloroquine, might lead to an increase in the amount of the reactive heme Fe^III^ in the vacuole, which is toxic for the parasite, and in this way limit the number of infected cells.

### 2.4. Soft X-ray absorption spectroscopy and spectromicros­copy of interfaces in renewable energy: fuel cells, lithium ion and lithium–air batteries   

**Figure 7:**
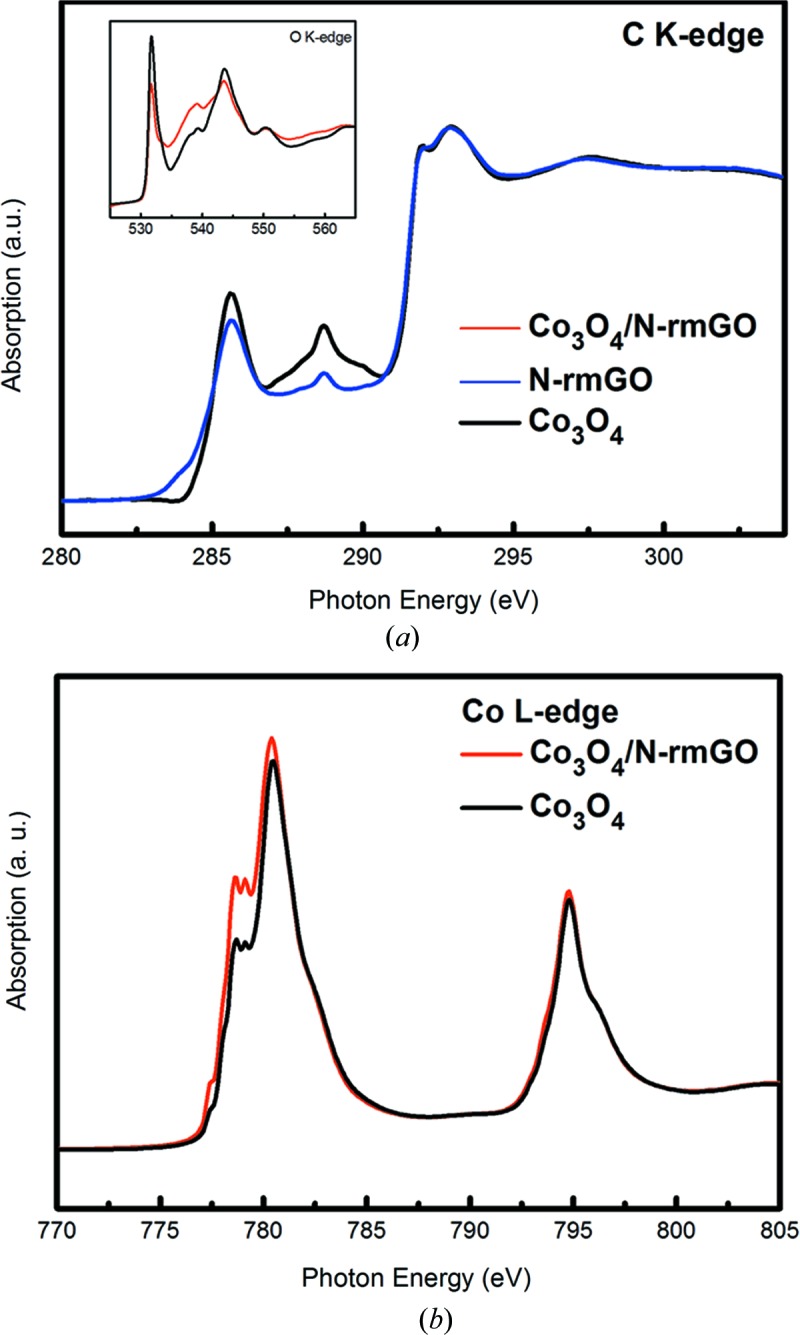
XANES spectra of (*a*) the C *K*-edge of graphene (N-rmGO) and Co_3_O_4_-coated graphene (Co_3_O_4_/N-rmGO), and at the O *K*-edge of Co_3_O_4_ and Co_3_O_4_-coated graphene (inset); (*b*) the Co *L*-edge of Co_3_O_4_ and Co_3_O_4_-coated graphene. The chemical bonding between Co_3_O_4_ and graphene in Co_3_O_4_-coated graphene has been demonstrated by the spectroscopic difference between free-standing Co_3_O_4_ (and graphene) and the hybrid (Co_3_O_4_-coated graphene). The enhanced 288 eV peak at the C *K*-edge in the hybrid highlights the possibility of a C—O—Co bond in the hybrid (Liang *et al.*, 2011 ▶).

**Figure 8:**
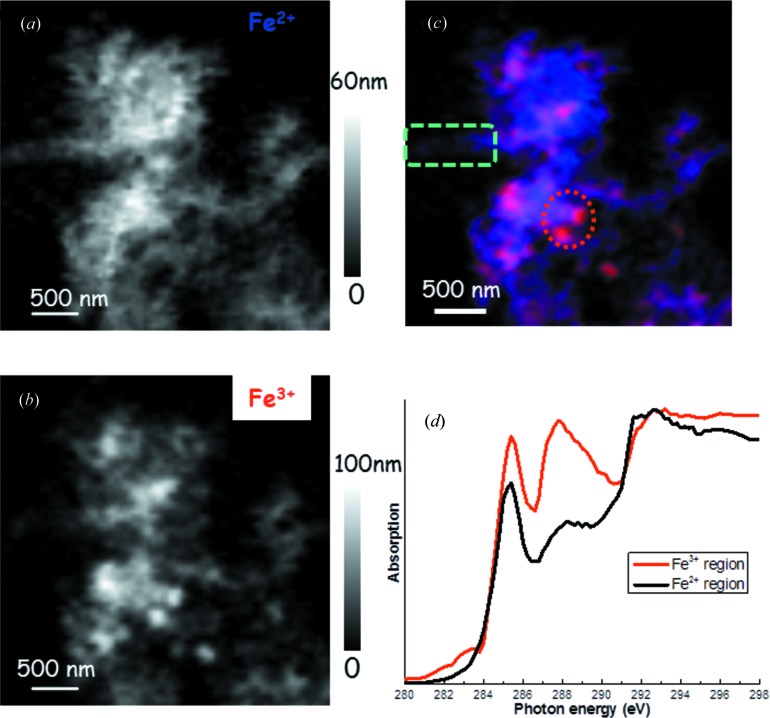
STXM chemical maps of partially charged LiFePO_4_–graphene for visualizing the Fe valance distribution: (*a*) Fe^2+^ and (*b*) Fe^3+^. The grey scale bars show the thickness in the maps. (*c*) The colour composite map of Fe^2+^ (blue) and Fe^3+^ (red). Selected Fe^2+^ and Fe^3+^ regions are highlighted by the rectangular and circular boxes, respectively. (*d*) C *K*-edge XANES spectra from the selected areas as displayed in (*c*). It is clear that the fast reaction region (Fe^3+^) has a much stronger 288 eV peak which could indicate a stronger LiFePO_4_–graphene interaction (Zhou *et al.*, 2013 ▶).

**Figure 9:**
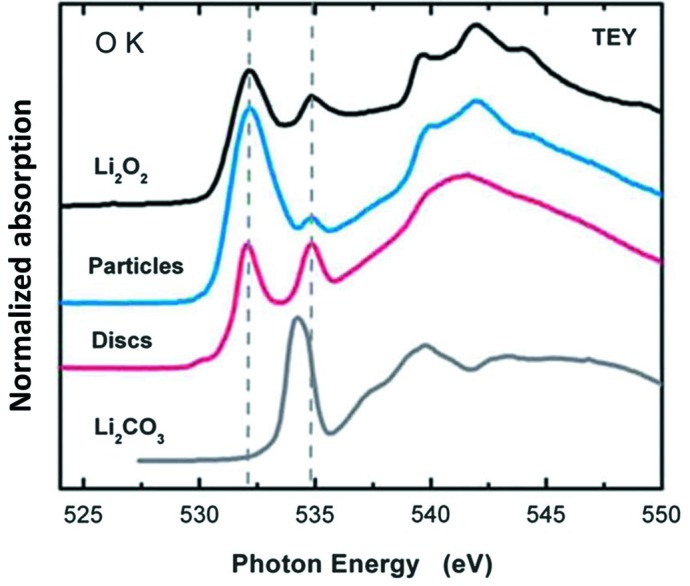
XANES spectra at the O *K*-edge of Li–air-discharged product with disc-like and particle-like morphology, and standard Li_2_O_2_ and Li_2_CO_3_. The electronic structure dependence on morphology is shown by the spectroscopic difference. The particle-like discharged product is closer to Li_2_O_2_ (Gallant *et al.*, 2013 ▶).

Deeper understanding of the interface (chemical and electronic structure) of active electrode materials with the electrolyte, and the interface between components within hybrid active electrode materials are crucial for the development of better electrochemical energy conversion devices [fuel cells (Liang *et al.*, 2011 ▶) and artificial photosynthesis for solar fuels] and energy storage devices [for instance batteries (Wang *et al.*, 2012 ▶; Zhou *et al.*, 2012 ▶)]. XANES spectroscopy can supply detailed information on the electronic structure and the local chemistry of the absorbing atom. With soft X-rays, XANES can supply information simultaneously on the surface with electron yield (probing depth of 5–10 nm) and subsurface with fluorescence yield (probing depth of 100 nm); such information is perfect for studying the surface and interface of battery materials even under *in operando* conditions. Further, Zhou *et al.* (2013 ▶) and Wang *et al.* (2013 ▶) showed that scanning transmission X-ray microscopy (STXM), based on the X-ray absorption process, has a chemical contrast mechanism that allows for imaging at the nanoscale, which can perfectly correlate performance and the structure variation in novel materials. Soft X-ray XANES at the C *K*-edge and the transition metal *L*-edge can identify the strong chemical bonding nature in graphene or carbon nanotubes along with supported novel inorganic hybrid nanostructures which make super-active nonprecious metal fuel cell catalysts (Liang *et al.*, 2011 ▶), as seen in Fig. 7 ▶, and battery electrode materials with greatly improved performance (Wang *et al.*, 2012 ▶, 2013 ▶; Zhou *et al.*, 2012 ▶, 2013 ▶). Furthermore, Zhou *et al.* (2013 ▶) and Wang *et al.* (2013 ▶) showed that the chemical bonding strength in the inorganic nano-hybrid within a single graphene sheet can be imaged using C *K*-edge STXM and the bonding variation can be correlated to the electrochemical activity variation by STXM at the metal *L*-edge, as seen in Fig. 8 ▶. It shows that stronger chemical bonding in the nano-hybrid favours faster electrochemical reaction. In addition to fuel cells and lithium ion batteries, a lithium–air battery is another alternative solution for sustainable future energy owing to its high theoretical energy density. However, from the practical point of view, it faces a lot of challenges such as low cycle efficiency, low cycle life and low power output. A fundamental understanding of the reaction process in this type of battery is therefore even more critical than for other types of energy devices. The sluggish lithium–air battery charging reaction (oxygen evolution reaction) has been observed to be affected by the surface structure (determined by XANES as seen in Fig. 9 ▶) of the discharge product (Gallant *et al.*, 2013 ▶).

### 2.5. *In situ* monitoring and validation of a uranium mill tailings management facility design   

Uranium ore containing elevated concentrations of arsenic (up to 2%) is processed at the Cameco Corporation (Key Lake mill) and Areva Canada (McLean Lake mill) operations in the Athabasca Basin in northern Saskatchewan. This region of northern Canada is responsible for more than 30% of the world production of uranium. A major concern of uranium mining operations, as well as provincial and federal regulators, is the risk that significant amounts of arsenic may pose to surrounding lakes and waters over tens of thousands of years after their disposal in the mills tailings management facilities (TMF). Arsenic contained within uranium mill tailings is the primary contaminant of concern in terms of potential to affect downstream receptors. The regulatory limit of arsenic is set at 5 mg l^−1^ for tailings pore water with an action level set at 2 mg l^−1^.

A majority of the arsenic is extracted from the ore during the mill leaching process with the soluble arsenic ultimately reported as a contaminant in downstream purification circuit waste solutions. Generally, the arsenic is removed from the acidic hydrometallurgical solution (raffinate) by precipitation with ferric iron and slate lime, producing low-solubility ferric arsenates. In order for a uranium mill operation to develop a solid plan for site remediation over several millennia, it is important to consider site characterization, risk assessment, and the selection of appropriate methods for clean-up and long-term monitoring. To properly develop an ecological and health risk assessment, questions surrounding bioavailability and long-range transport need to be addressed. Proper assessments can no longer be modelled on total contaminant concentrations, but instead must consider the chemical forms that are harmful. Synchrotron techniques are powerful methods for environmental research. Techniques such as XAS, XRD and X-ray fluorescence microprobe imaging can be used to identify the chemical species and oxidation states known to contribute to environmental or health risks. Additionally, the partitioning of unique chemical species between solid and aqueous phases can be measured and contaminant migration can be understood. Synchrotron techniques provide insight into a system by directly identifying and quantifying unique species within a complex matrix, with minimal sample modifications.

**Figure 10:**
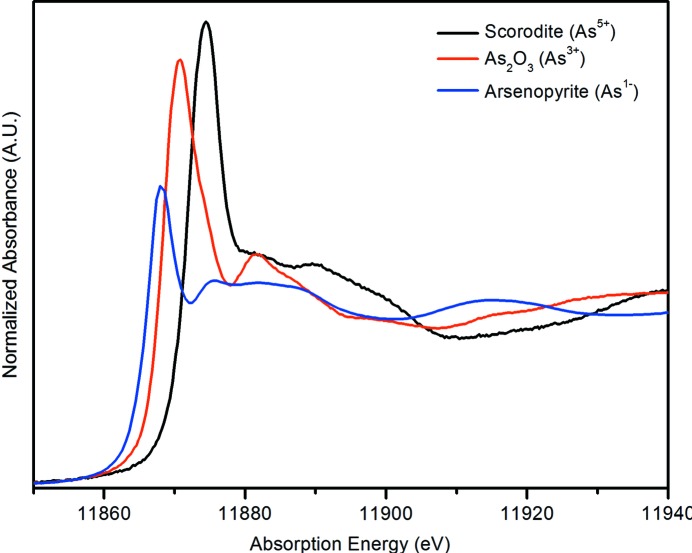
Arsenic *K*-edge XANES spectra of three standards showing how the resonance at ∼11875 eV shifts to higher energy with increasing oxidation state of the arsenic.

**Figure 11:**
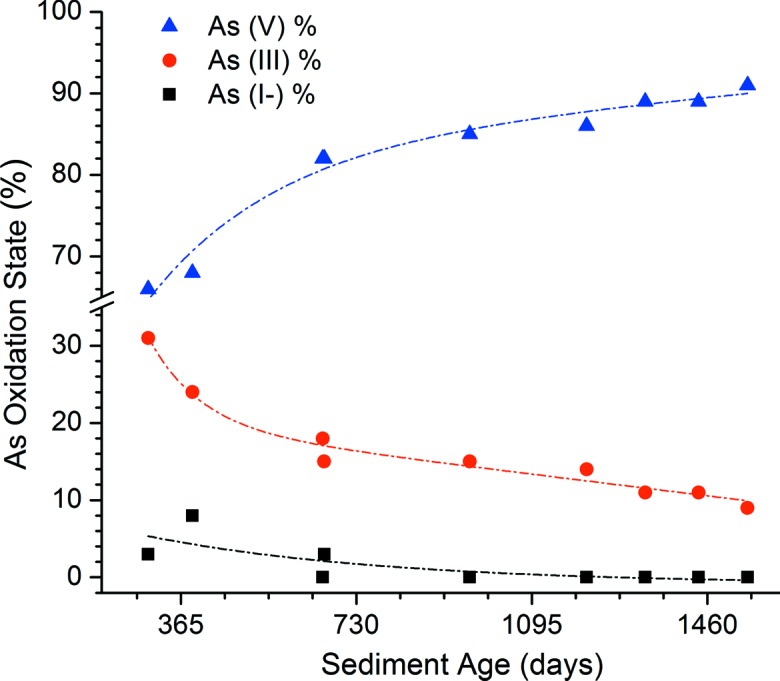
Relative arsenic oxidation state plotted against the tailings sediment age (Warner & Rowson, 2007 ▶).

Geochemical controls provide the means of controlling the long-term release of potential contaminants to the groundwater. The long-term stability of As within the tailings is controlled by the formation of Fe^III^–As^V^ mineral phases and complexes. Scorodite (FeAsO_4_·2H_2_O) is a poorly soluble mineral and is used to precipitate arsenic for deposition into the TMF. XANES spectroscopy is used to determine the oxidation state and the solid-state speciation of the arsenic after disposal in the tailings facility. Fig. 10 ▶ compares the As *K*-edge XANES spectra of three arsenic model compounds with different oxidation states (−1, +3, +5) and clearly shows that peak position of the white line (As 1*s* 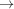 *np* type orbital) at ∼11874 eV is sensitive to oxidation state and can be used to determine relative amounts of arsenic species within complex mill tailings. Warner & Rowson (2007 ▶) reported the relative amounts of arsenic species found in borehole locations in the centre of the TMF at McLean Lake (Areva Canada). The samples represent a four-year period of exposure to the TMF environment. Fig. 11 ▶ shows both arsenide and arsenite phases are decreasing from highs of 8 and 31mol.% to lows of 0 and 9mol.%, respectively, over the 1544 day measurement period. Concurrently, the As^V^ oxidation state is increasing from 66 to 91% and the precipitated As is present as As^V^ in scorodite (Chen *et al.*, 2009 ▶), or as an arsenate precipitated on ferrihydrite (Essilfie-Dughan *et al.*, 2013 ▶). In addition, pore water sampling, carried out using a barge-mounted drill rig, at depth in the TMF, shows As^III^ concentrations exhibiting a time-dependent rise and fall in concentration, stabilizing after four years, at under 400 µg l^−1^ (data not shown). With this kind of information on the species deposited and its behaviour in TMF as a function of time, it is now possible for mining companies to monitor and control the release of various toxic contaminants over extended time periods as part of a site decommissioning and remediation plan.

## 3. Conclusions   

Synchrotron research facilities and other LSFs are determined to make themselves an integral part of industrial R&D. A few case studies have been described in this paper; however, it is worth a mention that a number of other studies in different disciplines are carried out at various synchrotron facilities. Whether helping business, in particular SMEs through Science Link (or other such programs around the world) to create new products, assisting with sustainability issues, owing to their unique capability to examine and illuminate environmental challenges, or providing solutions for major health concerns, and increasing understanding of novel materials, the impact of LSFs will be tangible and impactful. The future is bright!

## Figures and Tables

**Figure 2:**
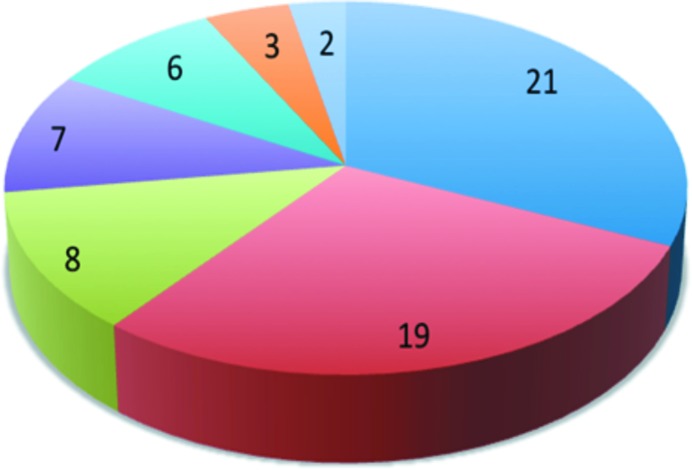
Distribution of projects by discipline: 21 projects devoted to construction and engineering, 19 to materials science and nanotechnology, 8 to life science and biotechnology, 7 to chemistry, 6 to environment and energy, 3 to agriculture and food science, and 2 to home and personal care.
